# Specialization of the Reiterated Copies of the Heterodimeric Integration Host Factor Genes in *Geobacter sulfurreducens*

**DOI:** 10.3389/fmicb.2021.626443

**Published:** 2021-03-02

**Authors:** Angel Andrade, Alberto Hernández-Eligio, Ana Lilia Tirado, Leticia Vega-Alvarado, Maricela Olvera, Enrique Morett, Katy Juárez

**Affiliations:** ^1^Departamento de Ingeniería Celular y Biocatálisis, Instituto de Biotecnología, Universidad Nacional Autónoma de México, Cuernavaca, Mexico; ^2^Departamento de Microbiología, Facultad de Medicina, Universidad Autónoma de Nuevo León, Monterrey, Mexico; ^3^CONACYT, Ciudad de México, Mexico; ^4^Instituto de Ciencias Aplicadas y Tecnología, Universidad Nacional Autónoma de México, Ciudad Universitaria, Ciudad de México, Mexico

**Keywords:** *Geobacter sulfurreducens*, transcriptome profile, pili, IHF, cytochrome

## Abstract

Integration host factor (IHF) is a widely distributed small heterodimeric protein member of the bacterial Nucleoid-Associated Proteins (NAPs), implicated in multiple DNA regulatory processes. IHF recognizes a specific DNA sequence and induces a large bend of the nucleic acid. IHF function has been mainly linked with the regulation of RpoN-dependent promoters, where IHF commonly recognizes a DNA sequence between the enhancer-binding region and the promoter, facilitating a close contact between the upstream bound activator and the promoter bound, RNA polymerase. In most proteobacteria, the genes encoding IHF subunits (*ihfA* and *ihfB*) are found in a single copy. However, in some Deltaproteobacteria, like *Geobacter sulfurreducens*, those genes are duplicated. To date, the functionality of IHF reiterated encoding genes is unknown. In this work, we achieved the functional characterization of the *ihfA-1, ihfA-2, ihfB-1*, and *ihfB-2* from *G*. *sulfurreducens.* Unlike the Δ*ihfA-2* or Δ*ihfB-1* strains, single gene deletion in *ihfA-1* or *ihfB-2*, provokes an impairment in fumarate and Fe(III) citrate reduction. Accordingly, sqRT-PCR experiments showed that *ihfA-1* and *ihfB-2* were expressed at higher levels than *ihfA-2* and *ihfB-1*. In addition, RNA-Seq analysis of the Δ*ihfA-1* and Δ*ihfB-2* strains revealed a total of 89 and 122 differentially expressed genes, respectively. Furthermore, transcriptional changes in 25 genes were shared in both mutant strains. Among these genes, we confirmed the upregulation of the *pilA*-repressor, GSU1771, and downregulation of the triheme-cytochrome (*pgcA*) and the aconitate hydratase (*acnA*) genes by RT-qPCR. EMSA experiments also demonstrated the direct binding of IHF to the upstream promoter regions of GSU1771, *pgcA* and *acnA*. PilA changes in Δ*ihfA-1* and Δ*ihfB-2* strains were also verified by immunoblotting. Additionally, heme-staining of subcellular fractions in Δ*ihfA-1* and Δ*ihfB-2* strains revealed a remarkable deficit of *c*-type cytochromes. Overall, our data indicate that at least during fumarate and Fe(III) citrate reduction, the functional IHF regulator is likely assembled by the products of *ihfA-1* and *ihfB-2*. Also, a role of IHF controlling expression of multiple genes (other than RpoN-dependent) affects *G. sulfurreducens* physiology and extracellular electron transfer.

## Introduction

Integration host factor (IHF) is a small heterodimeric DNA-binding and bending protein that promotes assembly of higher-order nucleoprotein structures involved in a variety of cellular processes, such as replication, transcription, gene expression, site-specific recombination, transposition, partition, transfer, and phage packaging (Rice et al., 1996; Ilves et al., 2004; Dorman, 2009; Groove, 2011). IHF was originally described in *Escherichia coli* as a factor required for the integration of bacteriophage lambda into the chromosome (Swinger and Rice, 2004). IHF belongs to the Nucleolar Associated Proteins (NAPs) family that comprises HU, Fis, and other proteins. HU is widely present in bacteria, while Fis, whose origin is the DNA binding domain of an ancestral alphaproteobacterial NtrC Enhancer-Binding Protein (EBP) (Morett and Bork, 1998), and IHF are mainly restricted to proteobacteria. Nonetheless, IHF has been found in some other bacterial clades, eukaryotic organisms, and viruses (Kamashev et al., 2017; Groove, 2011). The canonical structure of IHF consists of two paralogous subunits, IHFα and IHFβ, encoded by the unlinked *ihfA* and *ihfB* genes (Haluzi et al., 1991; Weisberg et al., 1996). Among other roles, IHF is required for the activation of genes transcribed by RpoN-containing RNA polymerase (Cannon et al., 1990; Hoover et al., 1990; de Lorenzo et al., 1991), including those participating in nitrogen fixation in *Klebsiella pneumoniae*, alginate synthesis in *Pseudomonas aeruginosa*, regulation of flagella genes in *Caulobacter crescentus*, toluene degradation in *Pseudomonas putida*, and cell division in *Desulfovibrio vulgaris* Hildenborough (Cannon et al., 1990; Santero et al., 1992; Calb et al., 1996; Delic-Attree et al., 1996; Muir and Gober, 2005; Fiévet et al., 2014). Upon binding, IHF bends the DNA, promoting the interaction between the bound upstream activator, EBP with the RpoN-RNA polymerase (Morett and Segovia, 1993). So far, IHF contribution has been evaluated only in bacterial species whose genome encodes a single copy of each IHF gene subunit (*ihfA* and *ihfB*). However, in some Deltaproteobacteria, including *Geobacter sulfurreducens*, multiple copies of these genes have been detected (Fiévet et al., 2014).

The *Geobacter* genus comprises species that are the predominant Fe(III)-reducing microorganisms in diverse subsurface environments (Holmes et al., 2004; Lovley et al., 2004). The insoluble nature of Fe(III) oxides requires that *Geobacter* species transfer electrons outside the cell via a long-range electron transfer mechanism, including a set of cytochromes plus extracellular conductive pili (Reguera et al., 2005; Smith et al., 2013). In addition, *G. sulfurreducens* can obtain energy by coupling the oxidation of organic compounds to carbon dioxide with the reduction of insoluble Fe(III), or electron transfer to electrodes, generating by this means electricity (Bond and Lovley, 2003; Lovley, 2006). Electron transfer to electrodes in *Geobacteraceae* has primarily been studied in *G. sulfurreducens*, because a genetic system and complete genome sequence are available (Coppi et al., 2001; Methé et al., 2003).

Although significant progress has been made in understanding the mechanism of electron transfer to Fe(III) in *G. sulfurreducens*, little is known about the regulatory mechanisms involved in controlling gene expression of the multiple products involved in transferring the electrons released from the central metabolism to the outside cell environment. Moreover, *G. sulfurreducens* encodes a high number of RpoN-dependent promoters and EBPs, leading us to hypothesize that IHF might play an important role in *G. sulfurreducens* physiology and extracellular electron transfer (EET).

In this study, we evaluated the expression of the four genes of *G. sulfurreducens* encoding IHF subunits, *ihfA-1*, *ihfA-2*, *ihfB-1*, and *ihfB-2*, and their contribution during growth under fumarate and Fe(III) as electron acceptors. Accordingly, subunits encoded by *ihfA-1* along with *ihfB-2* are predicted to conform the functional *G. sulfurreducens* IHF protein. Notably, gene deletion of *ihfA-1* or *ihfB-2* impacts PilA production and affects the *c*-type cytochrome content of the periplasm, inner, and outer membrane fractions. In addition, global transcription analysis by RNA-Seq of Δ*ihfA-1* and Δ*ihfB-2* strains, compared to the wild type strain, revealed a set of 89 and 122 differentially expressed genes, respectively. The RT-qPCR analysis, confirmed the altered expression of several genes, including GSU1771, *pgcA*, and *acnA*, and also the direct binding of IHF to such promoter regions was also demonstrated through EMSA experiments. Overall, this study corroborates the relevance of IHF as a global regulator that controls essential genes in EET, pili formation, and several cell processes in *G. sulfurreducens*.

## Materials and Methods

### Bacterial Strains, Plasmids, and Culture Conditions

*G. sulfurreducens* wild type strain DL1 (Caccavo et al., 1994) and its derivative *ihf* mutants (Table 1) were routinely cultured anaerobically in either acetate-fumarate or acetate-Fe(III) citrate medium, as previously described (Coppi et al., 2001). Anoxic sterile antibiotics (200 μgml^–1^ kanamycin, 50 μg ml^–1^ spectinomycin, or 10 μg ml^–1^ gentamicin) were added to acetate-fumarate plates during mutant strains selection. All *G. sulfurreducens* cultures were incubated at 30°C in an anaerobic chamber containing a mixture of 20% CO_2_, 80% N_2_. *E. coli* strains XL1-Blue and S17-1 (Table 1), were used for DNA manipulations and for conjugation experiments, respectively.

**TABLE 1:** List of strains and plasmid used in this work.

Name	Description	References
**Strains**		
***Geobacter sulfurreducens***		
DL1	Wild type	Caccavo et al., 1994
Δ*ihfA-1*	DL1 with *ihfA-1::km* mutation	This study
Δ*ihfB-1*	DL1 with *ihfB-1::km* mutation	This study
Δ*ihfA-2*	DL1 with *ihfA-2* deletion	This study
Δ*ihfB-2*	DL1 with *ihfB-2::Gm* mutation	This study
*E. coli*		
XL1-Blue	*recA1 endA1 gyrA96 thi-1 hsdR17c [F′ proAB lacIq ZΔM15 Tn10]*	Stratagene
S17-1	*recA pro hsdR RP4-2-Tc::Mu-Km::Tn7*	Simon et al., 1983
**Plasmids**		
pCR2.1 TOPO	Subcloning vector, ampicillin resistance and kanamycin resistance	Invitrogen
pTOPO HA	pCR2.1 TOPO with *ihfA-1* gene	This study
pTOPO HB	pCR2.1 TOPO with *ihfB-1* gene	This study
pK18mobsacB	*sacB* suicide vector	Simon et al., 1983
pK18mobsacB-ihfA2del	pK18mobsacB with flanking regions of *ihfA-2*	This study
pET19b	Bacterial expression vector, ampicillin resistance	Novagen
pETihfA1	pET19b with *ihfA-1* gene	This study
pTrc99a-FF4	Modified from pTrc99a without *Nde*I site and *Nco*I site converted to *Nde*I site	Ohnishi et al., 1997
pTrcihfB1	pTrc99a-FF4 with *ihfB-1* gene	This study
pATBis	pTrcihfB1 with *ihfA-1* gene cloned in operon with *ihfB-1*	This study
pATBis2	pATBis with *ihfA-1* gene cloned in operon with *ihfB-2*	This study
pRG5.1	Bacterial expression vector	Kim et al., 2005
pBBR1MCS-2	Bacterial cloning vector, kanamycin resistance	Kovach et al., 1994
pBSL141	Bacterial cloning vector, gentamicin resistance	Alexeyev et al., 1995
pRG5.1ihfB-2	pRG5.1 with *ihfB-2* gene	This study

### *In silico* Analysis of IHF Proteins

IHF protein identity analyzes were performed using the BLAST program^1^. Sequence alignments were performed using Clustal W^2^.

### DNA Manipulation

Plasmid purification, PCR product purification, and gel extractions were performed using the following kits: the QIAprep, Spin Miniprep Kit, the Qiagen Plasmid Midi Kit, the QIAquick PCR Purification Kit (Qiagen). Ligation reactions, *E. coli* transformations, and other routine DNA manipulations were carried out according to the methods outlined by Sambrook et al. (1989). Restriction enzymes and T4 DNA ligase (Thermo Scientific). *G. sulfurreducens* genomic DNA was extracted using the MasterPure complete DNA and RNA purification kit (Epicenter).

### RNA Purification and sqRT-PCR

Total RNA was isolated from *G. sulfurreducens* DL1 strain cultivated under two different conditions, using Fe(III) citrate or fumarate as the electron acceptor. Briefly, cultures were centrifuged at 4°C for 15 min, and pellets were flash frozen and stored at –80°C. Total RNA was isolated from mid-log-phase cultures using RNeasy Mini kits (Qiagen). Quality of total RNA was assessed by agarose-formaldehyde gel electrophoresis, and the concentration was determined using a NanoDrop 2000c (Thermo Fisher Scientific). By each RNA extraction, DNase treatment was carried out using DNase I, free RNase (Thermo Scientific). The reactions were cleaned up by the RNeasy Mini kit (Qiagen). Total RNA was measured again to start the following steps with the same concentration. cDNA was generated by reverse transcription using 200 units of SuperScript III reverse transcriptase (Invitrogen) and a hexamer random primer (5′NNNNNN3′) under the following program: 25°C for 10 min, 42°C for 50 min, 70°C for 10 min. The cDNA obtained was quantified by NanoDrop. The cDNA was amplified for semi-quantitative PCR reaction using 120 ng of cDNA, and specific oligonucleotides to the four *ihf* genes (Supplementary Table 1), using 1 unit of Phusion DNA polymerase (Thermo Scientific) under the following conditions: 1 cycle, 98°C for 30 seg; 26 cycles of 98°C for 10 seg, 60°C for 30 seg, 72°C for 15 seg, and finally one last extension cycle at 72°C for 5 min. At the reaction cycles 14, 20, and 26 a sample was removed and stored at 4°C. Specific pair primers to *r16S* (Supplementary Table 1) were used as positive control (Supplementary Figure 1). Finally, all PCR samples were purified by the MiniElute PCR purification Kit (Qiagen). And, the DNA was quantified by capillary electrophoresis using the Agilent Bioanalyzer 2100, DNA 1000 chip (Agilent Technologies).

### Construction of IHF Mutants

Single gene disruption of *G. sulfurreducens* DL1 *ihfA-1, ihfB-1*, and *ihfB-2* (*gsu1746*, *gsu1521*, and *gsu2602*) was achieved by the recombinant PCR and single-step recombination method (Murphy et al., 2000; Lloyd et al., 2003). All oligonucleotides sequences are placed into Supplementary Table 1. To disrupt the *ihfB-1* gene, a 2.09 kb DNA fragment was constructed by PCR in which 0.21 kb of the *ihfB-1* coding sequence (codons 8−88) were replaced with the kanamycin resistance cassette (kan^r^) of pBBR1CS-2 (Table 1). This fragment consisted of 449 bp of upstream sequence plus the first 22 bp of the *ihfB-1* gene (oligonucleotides HFBP1FW and NHFBP1REV), followed by the 1.1 kb from the kanr cassette (oligonucleotides NMUTBFW and NMUTBRV), and the last 43 bp of the *ihfB-1* gene plus 479 bp of downstream sequence (oligonucleotides HFBP3FW and HFBP3REV). A similar fragment (2.07 kb) was constructed to disrupt the *ihfA-1* gene. This fragment consisted of 485 bp of upstream sequence plus the first 39 bp of the *ihfA-1* gene (oligonucleotides NHFAP1FW and NHFAP1REV), followed by the 1.1 kb from the kan^r^ cassette (oligonucleotides NmutAFW and NmutARV), and the last 49 bp of the *ihfA-1* gene and 484 bp of downstream sequence (oligonucleotides NHFAP3FW and HFAP3REV).

To disrupt the gene *ihfB-2*, a 1.7 kb DNA fragment was constructed by PCR in which 0.23 kb of the *ihfB-2* coding sequence (codons 8−86) were replaced with the gentamicin resistance cassette (gm^r^) of pBSL141 (Table 1). This fragment consisted of 492 bp of upstream sequence together with the first 22 bp of the *ihfB-2* gene (oligonucleotides P1B2Fw and P1B2Rv), followed by the 0.7 kb from the gmr cassette (oligonucleotides P2B2HFw and P2B2HRv), plus the last 34 bp of the *ihfB-2* gene and 455 bp of downstream sequence (oligonucleotides P3B2Fw and P3B2Rv). *G. sulfurreducens* electrocompetent cells were prepared as previously described (Coppi et al., 2001), and transformed with the corresponding purified PCR fragment. A single kanamycin-resistant or gentamicin-resistant colony was selected and tested for the insertion of the cassette by PCR (Supplementary Figure 2).

To delete the gene *ihfA-2* (*gsu2120*), we used the markerless deletion method previously described (Chan et al., 2015). The flanking regions (871 bp upstream and 637 bp downstream) of *ihfA-2* were amplified with the primer sets ihfA2Fw1/ihfA2Rev2 and ihfA2Fw3/ihfA2Rev4 using *G. sulfurreducens* DL1 genomic DNA as the template and Phusion High-Fidelity DNA Polymerase (ThermoScientific). The flanking regions of *ihfA-2* were joined in a second round of PCR, digested with *Bam*HI and *Sal*I, and then ligated into the same sites in pK18mobsacB (Table 1). The resulting plasmid, pK18mobsacB-ihfA2del, was transformed into *E. coli* conjugative donor strain S17-1 to conjugate into the *G. sulfurreducens* recipient. One milliliter of fully grown *G. sulfurreducens* acetate-fumarate culture was pelleted on top of 1 ml of S17-1 culture carrying the sacB-carrying plasmid, mixed on top of a 0.22 μm-pore-size filter resting on acetate-fumarate agar plates in an anaerobic chamber, and incubated for 4 h before streaking the mixture onto acetate-fumarate plates with 200 μg ml^–1^ of kanamycin. This procedure selected *G. sulfurreducens* culture with pK18mobsacB-ihfA2del integrated into either flanking region of the gene since the plasmid cannot replicate in *G. sulfurreducens*. A scarless gene deletion mutant was selected on acetate-fumarate plates containing 10% sucrose and confirmed using PCR with primers flanking the deletion site (Supplementary Figure 2).

### Complementation of Δ*ihfB-2* Mutant

The *ihfB-2* gene was amplified using B2ECOFW and B2BAMRV oligonucleotides (Supplementary Table 1), Platinum Taq polymerase (Invitrogen), and *G. sulfurreducens* DL1 chromosomal DNA as template. The amplified fragment, flanked with *Eco*RI/*Bam*HI restriction sites, was double digested and cloned into the same sites of the pRG5.1 plasmid (Kim et al., 2005). The resulting plasmid containing the *ihfB-2* gene was sequenced and called pRG5.1ihfB-2. The plasmid was electroporated into Δ*ihfB-2* mutant strain and the resulting spectinomycin resistant colonies were screened for the plasmid’s presence by isolation of plasmid DNA followed by digestion with restriction enzymes.

### Cytochrome c Content

The membrane fractions of *G. sulfurreducens* DL1, Δ*ihfA-1*, and Δ*ihfB-2* strains, were isolated as previously described (Kim et al., 2005). Briefly, outer membrane-enriched fractions were prepared by treating crude membranes with a sarkosyl (sodium N-laurylsarcosinate) solution (1%, wt/vol) to extract inner membrane proteins. Five microgram of periplasmic, inner, and outer membrane proteins were separated by tris-tricine denaturing polyacrylamide gel electrophoresis, and *c*-type cytochromes were detected by heme staining with N,N,N,N-tetramethylbenzidine (Thomas et al., 1976; Francis and Becker, 1984). The total protein amount at each cellular fraction was observed by Coomassie staining. The proteins were visualized with Gel Doc DZ imager (Bio-rad).

### Immunoblot Analysis

Protein extraction from *G. sulfurreducens* DL1, Δ*ihfA-1*, and Δ*ihfB-2* strains was conducted as previously reported (Hernández-Eligio et al., 2020). Afterward, 1 μg of total protein per sample was incubated with PAGE-Buffer and boiled for 10 min before separation on a 15% SDS-PAGE. After, proteins were transferred to nitrocellulose membranes (Merck-Millipore) for immunoblot analysis using rabbit polyclonal antibodies raised against *G. sulfurreducens* PilA (Yi et al., 2009). Blots were blocked with 3% BSA in PBS overnight at 4°C and then incubated with a 1/1,000 dilution of primary antibody for 4 h at room temperature, washed with PBS, and incubated with a 1/5,000 dilution of goat anti-rabbit alkaline phosphatase-conjugated secondary antibody for 2 h at room temperature. After being washed, blots were developed with 1-Step NBT/BCIP substrate solution following manufacturer’s instructions (Thermo Scientific).

### Analytical Techniques

Protein contents were estimated with the Bradford method with bovine serum albumin as the standard (Biorad). Fe(II) concentrations were determined by the ferrozine assay (Lovley and Phillips, 1988).

### Expression and Purification of IHF Heterodimer

To express and purify an IHF heterodimer composed by subunits IHFα1 and IHFβ2, we constructed the pATBis2 plasmid (Table 1). First, *ihfA-1* and *ihfB-1* genes were amplified by PCR using *G. sulfurreducens* DL1 chromosomal DNA, Platinum taq Polymerase (Invitrogen), and PETAFW, PETARV, PETBFW, and PETBREV oligonucleotides (Supplementary Table 1). PCR products were cloned directly into pCR2.1 TOPO (Invitrogen), giving rise to pTOPO HA (*ihfA-1*) and pTOPO HB (*ihfB-1*). The *ihfA-1* gene was released from pTOPO HA by digestion with *Nde*I and *BamH*I, and cloned into a similarly digested pET19b plasmid given rise to pETihfA1. The *ihfB-1* gene was released from pTOPO HA by digestion with *Nde*I and *Hind*III, and cloned into a similarly digested pTrc99aFF4 plasmid given rise to pTrcihfB1. Then, *ihfA-1* gene fused with a sequence encoding six histidine codons was released from pETihfA1 by digestion with *Xba*I and *Hind*III, and cloned into a similarly digested pTrcihfB1 given rise to pATBis. On the other and, *ihfB-2* was amplified by PCR with ihfB2fw and ihfB2rev oligonucleotides (Supplementary Table 1), *G. sulfurreducens* DL1 chromosomal DNA, and Phusion polymerase. The PCR product was phosphorylated with PNK (ThermoScientific). By inverse PCR, *ihfB-1* gene was removed from plasmid pATBis using pBisfw and pBisrev2 oligonucleotides (Supplementary Table 1) and ligated to *ihfB-2* fragment to generate plasmid pATBis2. pATBis2 plasmid was sequenced to confirm the presence of an intact *ihfB-2* and *ihfA-1* genes and transformed into *E. coli* XL1-Blue. Expression of the IHF heterodimer in strain XL1-Blue/pATBis2 was induced by the addition of IPTG (0.1 mM). After 5 h induction, protein purification was performed at 4°C under non-denaturing conditions using a Ni-nitrilotriacetic acid affinity chromatography (Qiagen). The eluted protein was dialyzed using a buffer (40 mM HEPES, 8 mM MgCl_2_, and 50 mM KCl) and concentrated using Ultracel 3K (Amicon) at 4°C and stored at the same temperature.

### RNA-Seq and Data Analysis

The *G. sulfurreducens* cells from DL1, Δ*ihfA-1*, and Δ*ihfB-2* strain were used for RNA-Seq analysis. All experiments were performed in duplicates. For each biological sample, total RNA samples were extracted using the RNeasy mini kit (Qiagen) and then they were examined with an Agilent 2100 Bioanalyzer and quantified using NanoDrop 2000c (Thermo Scientific).

The RNA-Seq was performed using RNAs extracted from two independent cultures of each strain during the exponential growth phase (OD_600_ = 0.3) in the NBAF medium (acetate-fumarate). Illumina sequencing was performed at the UUSMD (UNAM, Mexico). RNA was processed as a previously described (Hernández-Eligio et al., 2020). Libraries were sequenced on an Illumina Genome Analyzer IIx. Differential expression analysis was performed using three methods: edgeR, DESeq, and NOISeq (Anders and Huber, 2010; Robinson et al., 2010; Tarzona et al., 2011). EdgeR and NOISeq were performed by applying TMM as the normalization method (Robinson and Oshlack, 2010). To identify differentially expressed genes, we selected those with *p*-value were 0.05 and logFC of 2, for each method. Finally, we considered only the genes that appeared differentially expressed in the three methods as the best candidates. The functional annotation of differentially expressed genes regarding the affected pathways was obtained from Kyoto Encyclopedia of Genes and Genomes (KEGG) (Kanehisa and Goto, 2000), using R’s own scripts.

### Quantitative RT-PCR (qRT-PCR) Assay

A subset of genes was selected to validate the quality of sequencing data by qRT-PCR. For RNA extraction, the cultures were grown in NBAF medium at 30°C and the cells were collected at the exponential growth phase (OD_600_ = 0.3). The mRNAs were extracted using a RNeasy mini kit (Qiagen) and residual DNA was removed using DNase I (Thermo Scientific). cDNA synthesis was performed using RevertAid H Minus First Strand cDNA Synthesis kit (Thermo Scientific). Subsequently, the qRT-PCR was performed using a Maxima SYBR Green/ROXqPCR Master Mix (Thermo Scientific) in a 96-well plate with the Light-Cycler II (Roche). Gene-specific oligonucleotides used for qRT-PCR are indicated in Supplementary Table 1. The *recA* was used as an internal standard gene for PCR amplification (Holmes et al., 2005). Normalized fold changes of the relative expression ratio were quantified by the 2^–ΔΔCT^ method (Livak and Schmittgen, 2001). All experiments were performed in triplicates and their average values were calculated.

### DNA Electrophoretic Mobility Shift Assays

Fragments of the regulatory region of GSU2678 (334 bp), *pgcA* (391 pb), *acnA* (242 pb), GSU1072 (*IcIR*, 510 pb), and GSU1771 (195 bp) were amplified from *G. sulfurreducens* DLI chromosomal DNA by PCR with the corresponding oligonucleotides pair (Supplementary Table 1). A fragment containing the regulatory region, used as a negative internal control in the DNA binding reactions, was also obtained by PCR with IG_303F and IG_303R oligonucleotides (Hernández-Eligio et al., 2017). PCR products were purified using the QIAquick PCR Purification Kit (Qiagen). Binding reactions were performed by mixing 100 ng of each PCR product with 100 ng of the IG_303 fragment (negative control) and increasing concentrations of purified IHF complex, in a total volume of 20 μl of binding buffer (Martínez et al., 2014). Binding buffer contained 40 mM HEPES, 8 mM MgCl_2_, 50 mM KCl, 1 mM DTT, 0.05% Nonidet P-40, and 0.1 mg ml^–1^ BSA. Protein-DNA binding reactions were incubated at room temperature for 30 min and then electrophoretically separated in 6% non-denaturing polyacrylamide gels in 0.5X Tris-borate-EDTA buffer at room temperature. The DNA fragments were stained with ethidium bromide and visualized with a Gel Doc DZ imager (Bio-rad).

## Results and Discussion

### *G. sulfurreducens* Has Duplicated *ihfA and ihfB* Genes

**FIGURE 1:**
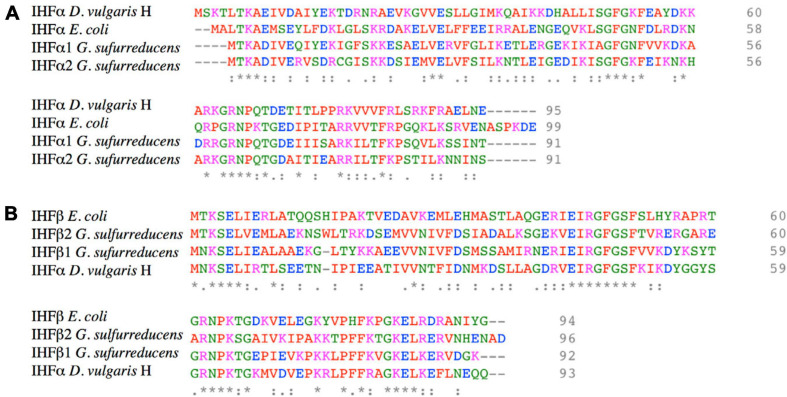
Amino acid sequence alignment of IHFα and IHFβ subunits of *G. sulfurreducens*, *E. coli*, and *D. vulgaris* H. **(A)** Protein sequence alignment of IHFα and IHFα’s subunits of *G. sulfurreducens*, *E. coli*, and *D. vulgaris* H. **(B)** Protein sequence alignment of IHFβ and IHFβ’s subunits of *G. sulfurreducens*, *E. coli*, and *D. vulgaris* H. The accessions numbers in NCBI database of IHFα1, IHFα2, IHFβ1, and IHFβ2 of *G. sulfurreducens* are AAR34895, AAR35496, AAR35123, and AAR35496, respectively. Colored amino acids have different properties: red are small or hydrophobic (including aromatic), blue is acid, magenta is basic, and green are hydroxyl, sulfhydryl, and amine. “*” indicate positions which have a single, fully conserved residue. “:” indicates conservation between groups of strongly similar properties, and “.” indicates conservation between groups of weakly similar properties.

*G. sulfurreducens* encodes four different subunits of the IHF heterodimer, two genes for the α subunit (*gsu1521*, named *ihfA-1;* and *gsu2120*, named *ihfA-2*), plus two for the β subunit (*gsu1746*, named *ihfB-1;* and *gsu2602*, named; *ihfB-2*). These genes are located in different *loci*, and their proteins present a moderate degree of conservation: IHFα1 is 63% identical to IHFα2 while IHFβ1 is 54% identical to IHFβ2. Among IHFα and IHFβ sequences, identity ranges from 38 to 31%. Amino acid sequence of IHFα1 and IHFα2 are 49 and 47% identical to IHFα of *E. coli*, respectively; while IHFβ1 and IHFβ2 are 47 and 48% identical with IHFβ of *E. coli*, respectively (Figure 1). These results suggest that the gene duplication present in the Deltaproteobacteria is very ancient but occurred after the separation of the Gamma and Deltaproteobacteria lineages. *D. vulgaris* H is the only Deltaproteobacteria in which the role of IHF has been studied; in this organism, IHF participates in the expression of the *orp* genes involved in cell division (Fiévet et al., 2014).

### Deletion of Δ*ihfA-1* or Δ*ihfB-2* Impairs *G. sulfurreducens* Respiration

**FIGURE 2:**
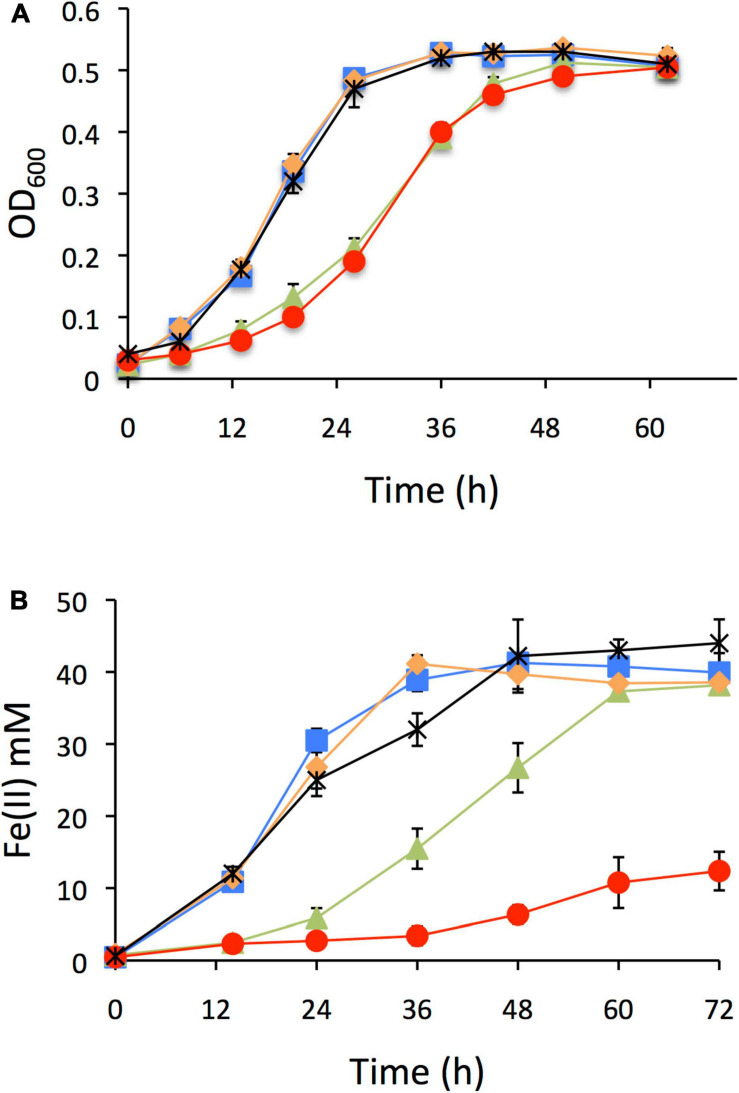
Characterization of *ihf* mutants of *G. sulfurreducens*. **(A)** Growth on fumarate as electron acceptor. **(B)** Soluble Fe(III) reduction. In all panels: DL1 line blue with square filled, Δ*ihfA-1* line brown with triangle filled, Δ*ihfA-2* line black with asterisk, Δ*ihfB-1* line orange with rhombi filled, Δ*ihfB-2* line red with circle filled.

In order to inquire into the functionality of each IHF copy and their possible physiological role, single null mutants of *ihfA-1*, *ihfA-2*, *ihfB*-*1*, and *ihfB-2* genes were constructed. Each strain was cultivated in a media containing different electron acceptors [fumarate or soluble Fe(III)]. In all cases, acetate was the main electron donor (Figure 2). Δ*ihfA-1* and Δ*ihfB-2* strains grew slower than the wild type strain, showing an extended lag phase; in contrast, no grow differences were observed between Δ*ihfA-2*, Δ*ihfB-1* and the wild type strains when fumarate was the sole electron acceptor (Figure 2A). Using soluble Fe(III) as a terminal electron acceptor, Δ*ihfA-1* strain showed an impaired Fe(III) respiration; and most significantly, Δ*ihfB-*2 strain stuck after a slight Fe(III) reduction (Figure 2B). These results suggest the relevant role of IHF in electron transfer to fumarate and soluble Fe(III). In contrast, Δ*ihfA*-*2* and Δ*ihfB*-*1* reduced soluble Fe(III) at a similar rate compared to the parental strain (Figure 2B). Moreover, indicate that under fumarate and soluble Fe(III) reduction conditions, the functional *G. sulfurreducens* IHF heterodimer is most likely assembled by IHFα1 and IHFβ2 subunits.

To confirm that the defective phenotype in growth and reduction of Fe(III) in Δ*ihfB-2* strain was due to the *ihfB-2* gene’s inactivation and not for a polar effect, we carried out the complementation of this mutant strain. Plasmids pRG5.1 or pRG5.1ihfB-2 were transformed into Δ*ihfB-2* strain. The Fe(III) reduction of Δ*ihfB-2* strain was complemented to wild-type levels in the presence of pRG5.1ihfB-2 plasmid but not the pRG5.1 vector control (Supplementary Figure 3).

### Expression Analysis of *ihf* Genes in Fumarate and Fe(III) as Electron Acceptors

**FIGURE 3:**
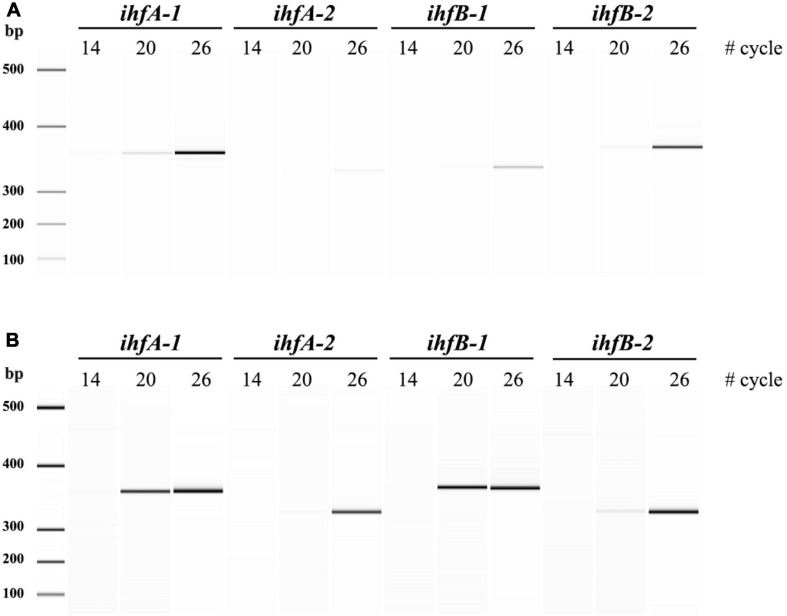
sqRT-PCR for *ihf*’s expression of *G. sulfurreducens*. **(A)** sqRT-PCR from acetate-fumarate. **(B)** sqRT-PCR from acetate-soluble Fe(III).

Following with the analysis of duplicated *G. sulfurreducens ihf* genes, we carried out a semi-quantitative RT-PCR assay to assess a differential gene expression between *ihfA-1*, *ihfA-2*, *ihfB*-*1*, and *ihfB-2* (Figure 3). The results reveal that the four *ihf* genes are transcribed under citrate Fe(III) and fumarate reducing conditions (Figure 3). Nevertheless, expression levels of *ihfA-1* were 35- and 17-fold higher compared to *ihfA-2*, while *ihfB-2* showed 9- and 5-fold higher expression compared to *ihfB-1* under fumarate and citrate Fe(III) reduction conditions, respectively. Thus, these significantly different expression data along with the observed phenotypes for the single mutant strains, suggests that the IHF heterodimer in *G. sulfurreducens* is composed by the IHFα1 and IHβ2 subunits.

### Whole Genome RNA-Seq Analysis of *G. sulfurreducens* DL1 vs. Δ*ihfA-1* and Δ*ihfB-2*

**FIGURE 4:**
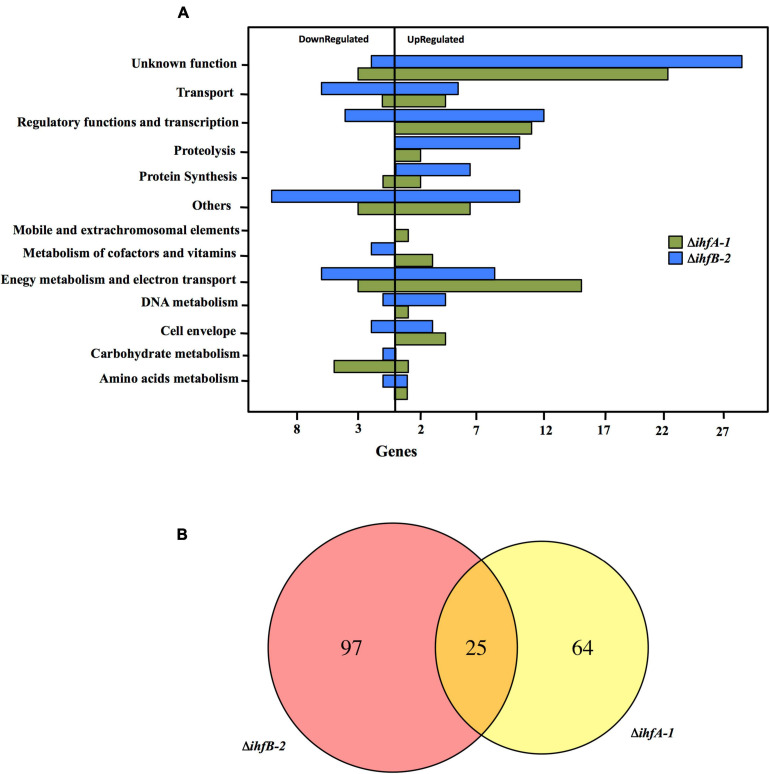
RNA-Seq analysis comparing transcriptome profiles of Δ*ihfA*-*1* and Δ*ihfB*-*2* strains compared to DL1. **(A)** Functional overview of the genes that were differentially expressed in Δ*ihfA-1* and Δ*ihfB-2* strains. **(B)** Venn diagram of the differential gene expression analysis from Δ*ihfA*-*1* and Δ*ihfB*-*2*.

To gain insight into the cellular processes that IHF regulates in *G. sulfurreducens*, we carried out RNA-Seq experiments to identify differential gene expression in Δ*ihfA*-1 and Δ*ihfB*-2 vs. DL1 strain in acetate-fumarate medium. We only considered genes that showed differential expression by the three methods evaluated in this study, DESeq, edgeR, and NOISeq (*p* < 0.05 and fold change >2). A total of 89 genes (73 upregulated and 16 downregulated) exhibited significant differential expression between DL1 and Δ*ihfA*-*1* strains (Supplementary Table 2), while 122 (85 upregulated and 37 downregulated) showed significant differential expression between DL1 and Δ*ihfB*-*2* strains (Supplementary Table 3). These genes were classified into 13 different functional classes (Figure 4A). The functional classes with the highest differentially expressed genes in the Δ*ihfA*-*1* strain compared to the wild type strain were: Unknown function (25); Energy metabolism and electron transport (18); and Regulatory functions and transcription (11). On the other hand, the functional classes with the highest differential expression genes in the Δ*ihfB*-2 compared to the wild type strain were: Unknown function (31); Others (20), Regulatory functions and transcription (16), and Energy metabolism and electron transport (14).

**FIGURE 5:**
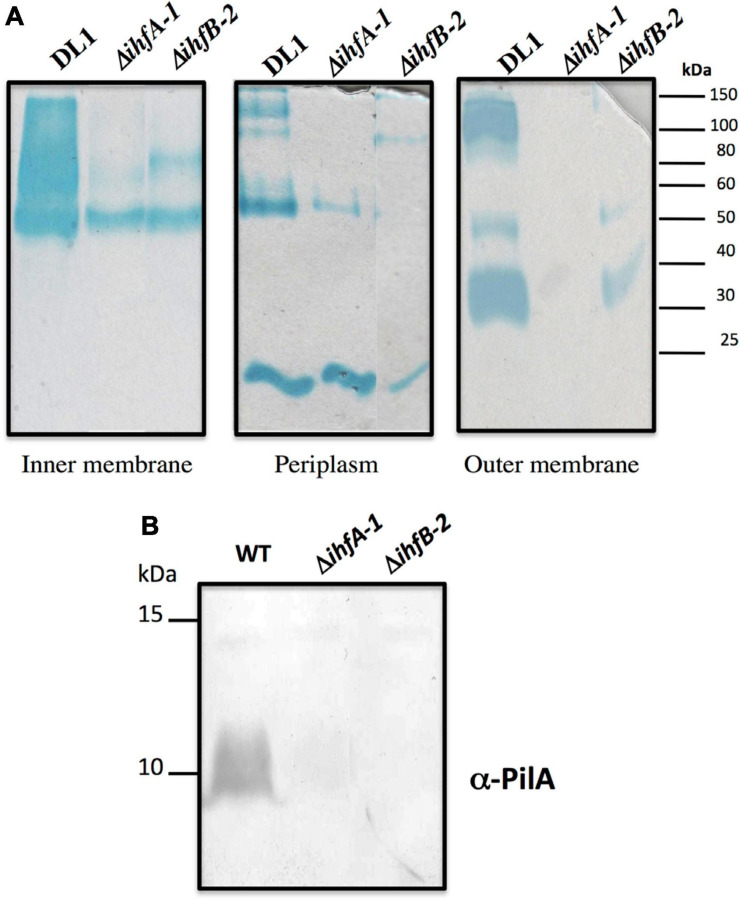
c-Type cytochrome and PilA content found in Δ*ihfA-1* and Δ*ihfB-2* strains. **(A)** Staining of *c-*type cytochromes from different cellular fractions. **(B)** Immunoblot analysis for PilA in total cells. The PageRuler Pre-stained Protein Ladder standard (Thermo Fisher Scientific) was used as a molecular weight. Full scans of the entire original gels are shown in Supplementary Figure 6.

RNA-Seq analyses also revealed that 25 genes were differentially expressed in both Δ*ihfA*-1 and Δ*ihfB*-2 strains (19 upregulated and six downregulated) compared to DL1 (Figure 4B and Table 2). One of these genes, *pgcA* (GSU1761), encodes the triheme-cytochrome PgcA whose expression and presence increase during growth with Fe(III) oxide compared to Fe(III) citrate (Ding et al., 2008; Aklujkar et al., 2013). The deletion of *pgcA* shows a severe reduction of Fe(III) and Mn(IV) oxides but not in Fe(III) citrate and NBAF (Tremblay et al., 2011; Zacharoff et al., 2017). While the addition of purified PgcA protein to wild type and Δ*pgcA* strains increases the rate of Fe(III) oxides reduction, 2- and 20-fold, respectively (Zacharoff et al., 2017). Other cytochromes with transcriptional changes in the Δ*ihfA*-1 strain compared to wild type were: *omcT* (GSU2503), *omcS* (GSU2504), GSU2937, GSU3228, GSU3232, GSU3233 (upregulated), and in the Δ*ihfB*-2 strain were: *ppcB* (GSU0364), *omcE* (GSU0618) (upregulated) and GSU1785, GSU2725, GSU2883, GSU3274 (downregulated). Accordingly, the heme stain of subcellular fractions of Δ*ihfA*-1 and *ihfB*-2 strains revealed an extensive lack of most periplasmic, inner and outer membrane associated cytochromes compared to DL1 strain (Figure 5A). This reduced cytochrome content was not due to differences in protein loading, as validated by Coomassie Blue staining (Supplementary Figure 4). The notoriously affected cytochromes content in OM enriched fraction from Δ*ihfA-1* and Δ*ihfB-2* strains, is also in agreement with the impaired soluble Fe(III) reduction previously observed in both strains (Figure 2B).

**TABLE 2:** List of genes with significant differential expression in Δ*ihfA-1* and Δ*ihfB-2* strains compared to the DL1 strain.

			Average *n*-fold change	
**Regulation**	**Locus ID**	**Common name**	**Δ*ihfA-1***	**Δ*ihfB-2***
Upregulated genes	GSU0050	HIRAN domain-containing protein	3.2276	2.6253
	GSU0216	Hypothetical protein	3.1211	3.1363
	GSU0469	Hypothetical protein	2.1078	3.3448
	GSU0654	Thiamin biosynthesis thiocarboxylate synthase, *thiF-1*	2.3445	3.0897
	GSU0725	Hypothetical protein	2.3397	4.05
	GSU0756	Methyl-accepting chemotaxis sensory transducer, *mcp40H-24*	2.1209	2.9501
	GSU0836	RNA polymerase-binding protein Rnk, *rnk-2*	3.6744	6.6063
	GSU1072	IclR family transcriptional regulator	2.8499	3.961
	GSU1771	DNA/RNA-binding protein	2.1474	3.2477
	GSU1943	PEP motif-containing protein exosortase substrate	2.9812	2.8307
	GSU2077	Hypothetical protein	2.4391	3.9434
	GSU2078	Rod shape determining protein RodA, *rodA*	2.1888	3.1208
	GSU2355	Hypothetical protein	2.4721	3.3426
	GSU2403	Hypothetical protein	2.757	3.3031
	GSU2410	ATP-independent chaperone, *hspA-2*	2.8468	6.53
	GSU2678	ATP-independent chaperone	3.5087	5.318
	GSU3514	Hypothetical protein	3.1357	2.4363
	GSU3545	Hypothetical protein	3.9183	4.3686
	GSU3546	Hypothetical protein	2.7054	2.3838
Downregulated genes	GSU0182	Murein lipoprotein	–2.0813	–2.2693
	GSU0846	Aconitate hydratase, *acnA*	–2.0336	–2.038
	GSU0847	Rubredoxin	–2.8882	–2.7882
	GSU0848	Ferredoxin, frx-5	–2.4185	–2.7748
	GSU1761	Lipoprotein cytochrome c, *pgcA*	–2.6794	–2.1816
	GSU1945	Repeat-containing protein	–2.1108	–3.2159

The *acnA* (GSU0846) gene was also downregulated in Δ*ihfA-1* and Δ*ihfB-2* strains compared to DL1 strain. It codes for aconitate hydratase I, an enzyme that catalyzes the isomerization of citrate to isocitrate via cis-aconitate. Thus, the reduced expression of *acnA* in Δ*ihfA-1* and Δ*ihfB-2* strains might exert a negative effect on the tricarboxylic acids metabolism delaying the growth of both mutant strains during fumarate reduction (Figure 2A). However, the contribution of the other two *G. sulfurreducens* genes encoding putative aconitase hydratases (GSU1660 and GSU2445) still needs to be clarified.

Another gene that was differentially expressed in the Δ*ihfA-1* and Δ*ihfB*-*2* strains compared to the parental strain was GSU1771 (upregulated in both mutant strains). It encodes a transcriptional regulator homologous to the *Streptomyces* antibiotic regulatory protein (SARP) that regulates secondary metabolism (Tremblay et al., 2011; Reguera and Kashefi, 2019). Disruption of *G. sulfurreducens* GSU1771 gene was previously reported to have a positive effect during Fe(III) oxides reduction, and this phenotype was associated with an increased expression of the gene encoding the structural pili protein, *pilA* (Tremblay et al., 2011). Therefore, we decided to evaluate the impact of *ihfA*-*1* and *ihfB*-*2* deletion over PilA production by immunoblot (Figure 5B). Notably, PilA was not detected in Δ*ihfA-1* and Δ*ihfB-2* strains, contrary to the parental strain (Figure 5B).

GSU1072 encodes a IclR family regulator, which is upregulated in the Δ*ihfA-1* and Δ*ihfB-2* strains. Proteins grouped in this family control genes whose products are involved in the glyoxylate shunt in Enterobacteriaceae, multidrug resistance, degradation of aromatics, and inactivation quorum-sensing signals, determinants of plant pathogenicity and sporulation (Molina-Henares et al., 2006). In *G. sulfurreducens*, GSU1072 controls the metabolism of hydrogen and transport of acetate by regulating the transcription of the Hya operon and the GSU1068 and GSU1070 genes (Mahadevan et al., 2008).

The expression of RpoN-dependent promoters requires efficient communication between enhancer proteins (EBPs) and bound promoter RpoN-RNAP holoenzyme, usually separated by 100–140 bp. Due to the large number of RpoN- dependent promoters and different EBPs in *G. sulfurreducens*, we hypothesized that IHF participates in the expression of a large number of genes. Genome-wide microarray transcriptional profiling of a RpoN over-expression strain revealed changes in the genome expression profile of 196 genes (Leang et al., 2009). Our RNA-Seq analysis showed that 19 and 7 genes with transcriptional changes in Δ*ihfB-2* and Δ*ihfA-1* strains, respectively (Supplementary Table 4), also have changes in the RpoN regulon (Leang et al., 2009). Moreover, we detected 20 and 10 differentially expressed genes in the Δ*ihfA-1* and Δ*ihfB-2* strains, respectively, containing putative RpoN-promoters in their regulatory region (Supplementary Table 5). These data suggest that genes with transcriptional change and predicted RpoN-promoters in the Δ*ihfA-1* and Δ*ihfB-2* strains might be direct targets of regulation by IHF and RpoN. Future studies are necessary to determine their regulatory mechanisms.

### qRT-PCR Gene Expression of Selected Genes

To corroborate our RNA-Seq results, some genes were selected for further validation through quantitative RT-PCR (qRT-PCR). Selected genes encode proteins involved in electron transfer (*pgcA*, GSU1785, and *pilA*), transcriptional regulators (GSU1771, *gnfR*, *gnfK*), central metabolism (*acnA*), and others (GSU0421, GSU2408, and *yedY*) (Table 3). Upregulation of GSU1771 and downregulation of *acnA* and *pgcA* in Δ*ihfA-1* and Δ*ihfB-2* strains compared with wild type was confirmed by qRT-PCR. Similarly, the expression of *fliM*, *gnfK*, *gnfR*, and GSU2408 was high in Δ*ihfA-1*, and low transcription of GSU1785 and GSU2723 observed in the RNA-Seq analysis of Δ*ihfB-2* strain was also confirmed by qRT-PCR. Furthermore, the low transcription of *pilA* gene in Δ*ihfA-1* and Δ*ihfB-2* strains was also corroborated (Table 3), being in agreement with our immunoblot results (Figure 5B), and with the higher expression of GSU1771 determined for both mutant strains compared to DL1 strain (Supplementary Tables 2,3). These data support the critical role of IHF in *G. sulfurreducens* physiology and EET.

**TABLE 3:** Expression of genes with relevant phenotype observed in RNA-seq experiments comparing Δ*ihfA-1* and Δ*ihfB-2* vs. DL1 strains by qRT-PCR.

Locus ID	Common name	Avg Δ*ihfA-1*/Avg DL1	Avg Δ*ihfB-2/*Avg DL1
GSU0846	Aconitate hydratase, *acnA*	0.408	0.288
GSU1771	DNA/RNA-binding protein	1.617	1.717
GSU1761	Lipoprotein cytochrome c, *pgcA*	0.091	0.305
GSU0421	Flagellar motor switch protein, *fliM*	2.713	ND
GSU0941	Nitrogen fixation sensor histidine kinase, *gnfK*	21.4	ND
GSU2822	Nitrogen fixation response regulator, *gnfR*	6.964	ND
GSU2408	Heat shock protein, Hsp20 family	4.387	ND
GSU1785	Cytochrome c	ND	0.532
GSU2723	Periplasmic sulfoxide reductase, *yedY*	ND	0.283
GSU1496	PilA protein, *pilA*^*a*^	0.054	0.045

*n-fold changes were calculated based on the 2^–ΔΔCT^ method (Livak and Schmittgen, 2001). Avg, Average and ND, not determined. ^*a*^Not determined by RNA-Seq but validated by RT-qPCR.*

### IHFβ2 Copurified With IHFα1

Our results suggest that the products of *ihfA*-*1* and *ihfB*-*2* genes assembly the functional IHF complex in *G. sulfurreducens*, regulating the transcription of several elements responsible for the transfer of electrons to soluble and insoluble acceptors such as cytochromes and pilin. To verify the interaction between IHFα1 and IHFβ2, both genes were cloned in tandem under the same promoter, while a sequence coding a histidine tag was added to *ihfA-1* (see section “Materials and Methods”). After protein induction in *E. coli*, we evaluated the capability of IHFα1 to retain the untagged IHFβ2, and co-elute from a Ni-NTA resin (Supplementary Figure 5). This pull-down assay corroborated the interaction between IHFα1 and IHFβ2. The interaction of IHFβ2 with IHFα1 was specific since no elution of IHFβ2 was observed in unbound and washed fractions. These results, along with our previous data, suggest that in *G. sulfurreducens*, IHFα1 and IHFβ2 compose the major IHF heterodimer.

### IHF Binds to the Promoter Regions of the *acnA, GSU1771, pgcA, GSU2678*, and *GSU1072* Genes

**FIGURE 6:**
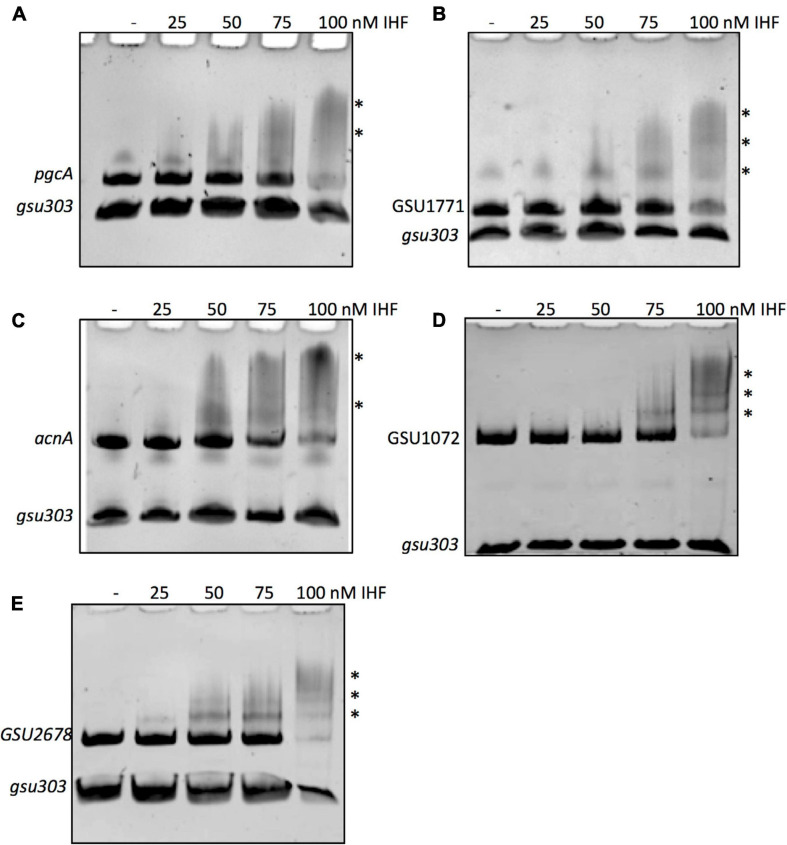
IHF binding to the DNA fragments of promoter regions was analyzed by competitive non-radioactive EMSAs. PCR-amplified and purified fragments of *pgcA* **(A)**, GSU1771 **(B)**, *acnA* **(C)**, GSU1072 (*IcIR*) **(D)**, GSU2678 **(E)** were incubated with increasing concentrations (0, 25, 50, 75, and 100 nM) of purified IHF complex (IHFα1/IHFβ2). As a negative control, a fragment containing the intergenic region *gsu303* was included in each DNA-binding reaction. The asterisks show the DNA-protein complexes.

To assess whether the IHF directly contributes to the expression of the *acnA*, GSU1771, GSU2678, *pgcA*, and GSU1072 (*IcIR*) genes, we evaluated the binding of IHF to their promoter regions by electrophoretic mobility shift assay (EMSA). DNA fragments containing the whole intergenic regions of *acnA* (242 bp), GSU1771 (195 bp), *pgcA* (391 pb), GSU1072 (510 bp), and GSU2678 (334 bp) were amplified by PCR and subjected to EMSA with purified IHF (IHFβ2/IHFα1). As shown in Figure 6, the interaction of IHF with all promoter regions was observed. These interactions were specific since no binding of IHF with the control DNA fragment was observed (*gsu303*). These results strongly suggest that the IHF heterodimer composed by IHFβ2 and IHFα1 directly affects the transcription of *acnA*, GSU1771, *pgcA*, GSU1072, and GSU2678 genes through the binding to their promoter regions, and suggests that the rest of the genes with transcriptional changes in both Δ*ihfA-1* and Δ*ihfB-2* strains could be directly regulated by IHF.

### Identification of IHF Binding Sites in *acnA, GSU1771, pgcA, GSU2678*, and *GSU1072* Promoter Regions

**FIGURE 7:**
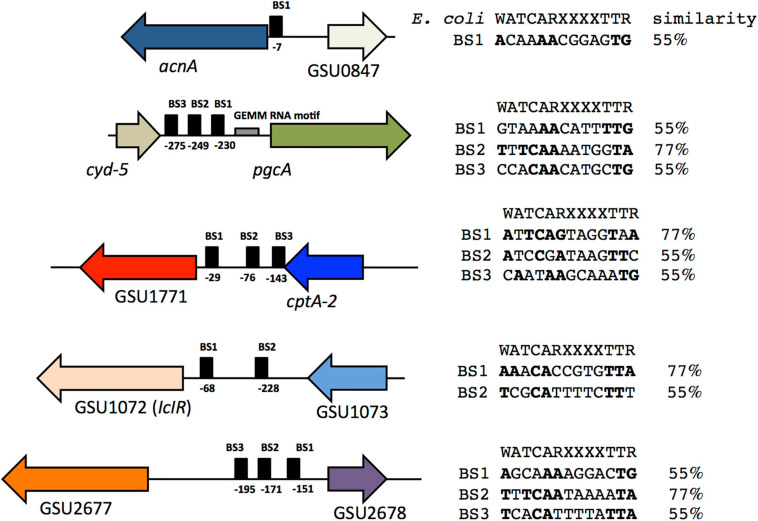
Putative IHF binding sites in the *acnA*, *pgcA*, GSU1771, GSU 2678, and GSU1072 (*IcIR*) promoter regions. The IHF *E. coli* binding site consensus sequence is shown. Conserved nucleotides are in bold and the percentage of similarity with respect to the consensus sequence is shown.

We carried out an *in silico* analysis of *acnA*, GSU1771, *pgcA*, GSU2678, and GSU1072 promoter regions to identify potential IHF binding sites that matched the consensus binding site 5′-WATCARXXXXTTR-3′ (W is A or T, R is A or G) of *E. coli* (Hales et al., 1994), using the Virtual footprinting program^3^ (Figure 7). *In silico* analysis suggest the presence of one potential IHF binding site within the *acnA* promoter region (BS1), located at position –7 from the start of translation. In the promoter region of *pgcA* there are three potential IHF binding sites (BS1, BS2, BS3), located at positions –230, –249, and –275 from the start of translation, upstream of the predicted GEMM RNA motif element (–64 to –170). In the promoter region of GSU1771 three binding sites were predicted (BS1, BS2, BS3), located at –29, –76, and –143, respectively, from the start of translation. On the other hand, in the regulatory region of GSU1072 (*IcIR*) two possible binding sites were predicted (BS1 and BS2), located at –68 and –228 upstream of the translation start. Finally, in the regulatory region of GSU2678, there were three putative binding sites (BS1, BS2, BS3), located at –151, –171, and –195 from the start of translation.

In *D. vulgaris* H, IHF regulates transcription of the *orp1* and *orp2* genes involved in cell division; each gene has one functional binding site for IHF, and they match the consensus IHF recognition sequence of *E. coli* (Fiévet et al., 2014). Together, the results suggest that some of the predicted IHF binding sites in the regulatory regions analyzed are functional in *G. sulfurreducens*. Site-directed mutation studies on these sites are necessary to confirm this hypothesis.

## Conclusion

In this work, we elucidated the role of IHF in electron transfer to fumarate, and soluble Fe(III) in *G. sulfurreducens*. To our knowledge, this is the first report where single *ihf* gene mutations strongly affect the cytochrome content in bacteria. In contrast to HU, another member of the DNABII family of DNA-binding proteins, IHF operates as a heterodimeric protein composed of an α and a β subunits; nevertheless, homodimers could conserve some functionality. Concerning that notion, heterologous expression *in trans* of *G. sulfurreducens ihfA*-*1* or *ihfB-1* (independent genes under *trc* promoter) severely affects *E. coli* growth (data not shown).

The sqRT-PCR results showed that, albeit at a different level, all four *ihf* genes are transcribed during fumarate and soluble Fe(III) reduction; so it is plausible to infer that more than one possible combination between the IHF subunits eventually can occur. For instance, in the absence of each of the two most abundant subunits (IHFα1 or IHFβ2), homodimeric or different complexes could occur. However, evaluation of stability/functionality of *G. sulfurreducens* IHF hetero- and homodimers requires to be evaluated. These results suggest that, at least under the evaluated conditions, *G. sulfurreducens* IHF heterodimeric complex is most likely composed of IHFα1 and IHFβ2. A plausible interpretation of the *ihf* duplicity could be related to the significant ammount of RpoN-dependent promoters in *G. sulfurreducens*.

## Data Availability Statement

The datasets generated for this study can be found in GEO repository, to review GEO accession GSE160901: https://www.ncbi.nlm.nih.gov/geo/query/acc.cgi?acc=GSE160901.

## Author Contributions

AA and AH-E contributed to the conceptualization, investigation, and formal analysis. KJ carried out the design, supervised, and coordinated the study. LV-A performed the RNA-Seq statistical analyses. AT and MO contributed to the plasmids construction for IHF expression and performed the EMSA assay. AA, AH-E, EM, and KJ wrote the manuscript. All authors contributed to the article and approved the submitted version.

## Conflict of Interest

The authors declare that the research was conducted in the absence of any commercial or financial relationships that could be construed as a potential conflict of interest.

## Footnotes

1 https://blast.ncbi.nlm.nih.gov/Blast.cgi

2 https://www.ebi.ac.uk/Tools/msa/clustalw2/

3 http://prodoric.tu-bs.de/vfp/vfp_promoter.php
