# Supplementary material for: Specialization of the Reiterated Copies of the Heterodimeric Integration Host Factor Genes in Geobacter sulfurreducens
Source: Front Microbiol. 2021 Mar 2;12:626443. doi: 10.3389/fmicb.2021.626443 (PMC7962754; doi:10.3389/fmicb.2021.626443)
Supplement: Supplementary file 1 [file Data_Sheet_1.pdf]

Supplementary Material

1 Supplementary Tables

SUPLEMENTARY TABLE 1 Oligonucleotides used in this work

SUPPLEMENTARY TABLE 2 Differentially expressed genes in the Δ*ihfA-1* strain as compared of the DL1 strain

SUPPLEMENTARY TABLE 3 Differentially expressed genes in the Δ*ihfB-2* strain as compared of the DL1 strain

SUPPLEMENTARY TABLE 5 List of differentially expressed genes in Δ*ihfA-1*, Δ*ihfB-2* and σ^{54} transcriptome (Leang et al., 2009)

SUPPLEMENTARY TABLE 5 List of differentially expressed genes in Δ*ihfA-1*, Δ*ihfB-2* strains with predicted σ^{54}-regulated promoter (Leang et al., 2009)

2 Supplementary Figures

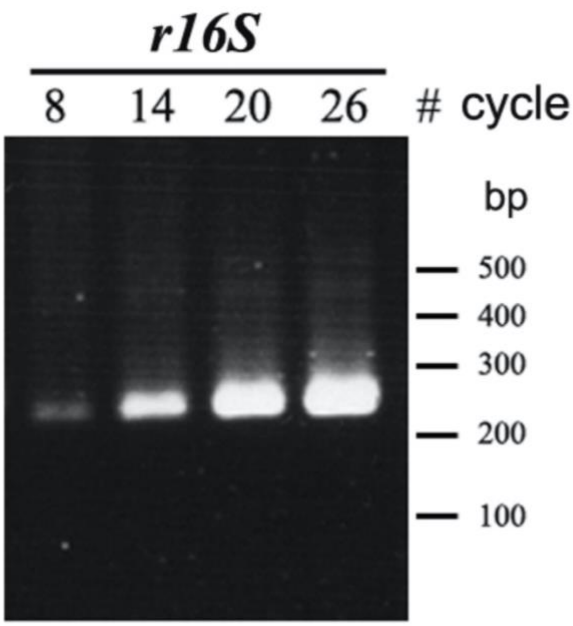

SUPPLEMENTARY FIGURE 1 sqRT-PCR of r16S used as a positive control in expression analysis of *ihf* genes. Specific oligonucleotides for r16S were used as control. Negative controls were performed without cDNA synthesis, in these cases no amplification was observed after analyze PCR final products (data no shown).

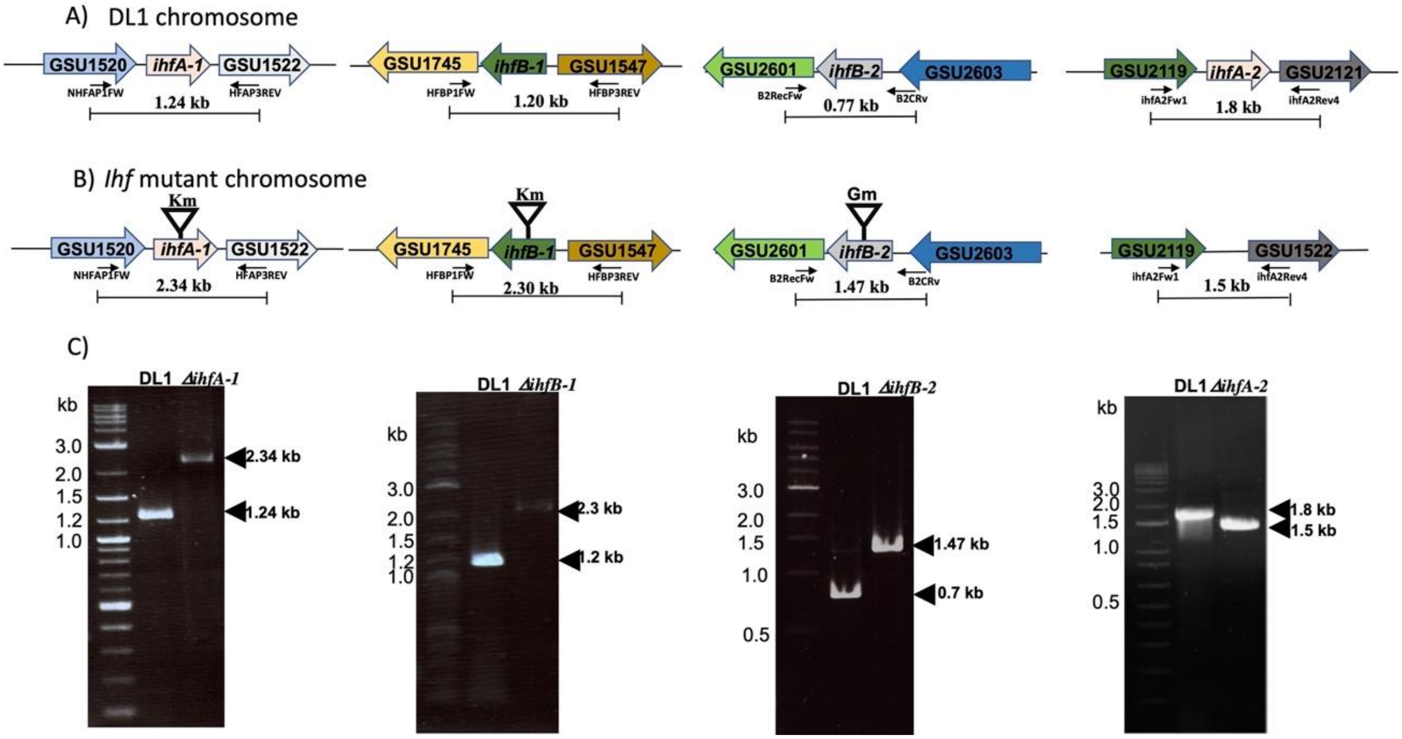

SUPPLEMENTARY FIGURE 2 Confirmation of the mutations in the *ihf´s* genes from *Geobacter sulfurreducens*. A) Organization of the *ihf`s* genes on DL1 chromosome. B) Reorganization of *ihf* locus after of the km or gm resistance gene insertion on *ihfA-1*, *ihfB-1* or *ihfB-2*, and *ihfA*-2 deletion. C) Confirmation by PCR of *ihf* mutants. The primer pairs used are enlisted in ST1, and the expected amplicons sizes are shows in A) and B). The arrowheads in C) indicate the expected DNA fragments.

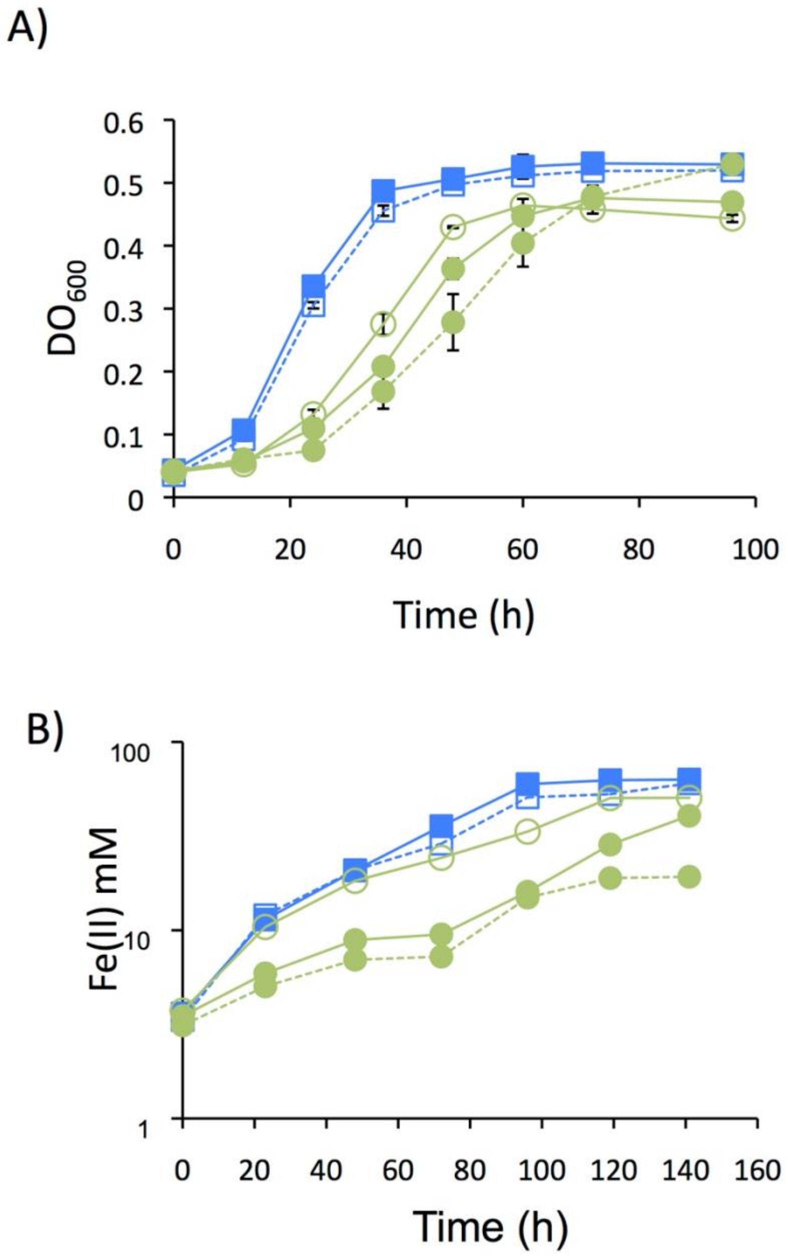

SUPPLEMENTARY FIGURE 3 Complementation of Δ*ihfB-2* mutant. A) Growth on fumarate as electron acceptor. B) Soluble Fe(III) reduction in *ihfB-2* complementation. DL1 blue line with squares filled, DL1/pRG5.1 dashed blue line with empty squares, Δ*ihfB-2* brown line with circle filled, Δ*ihfB-2*/pRG5.1 dashed brown line with circle filled and Δ*ihfB-2*/pRG5.1ihfB2 brown line with empty circle.

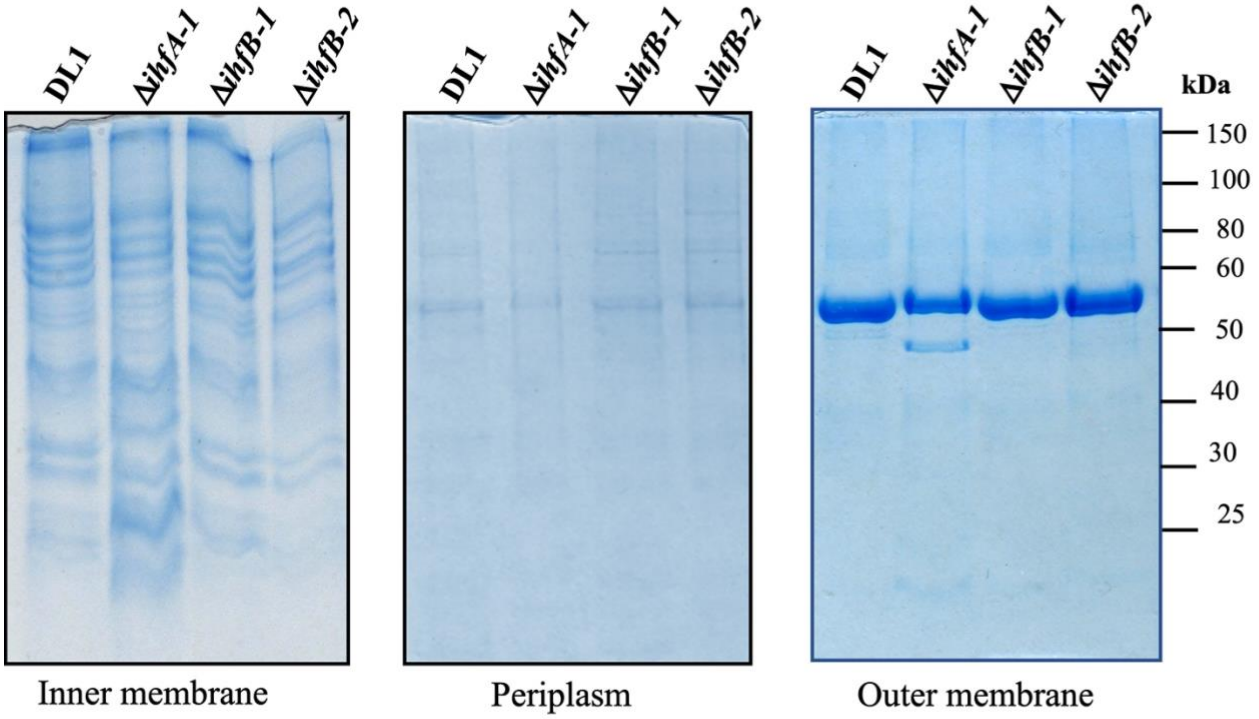

SUPPLEMENTARY FIGURE 4 Content of total protein from different cellular fractions of *ihf* mutants of *G. sulfurreducens*. The PageRuler Pre-stained Protein Ladder standard (ThermoScientific) was used as a molecular weight.

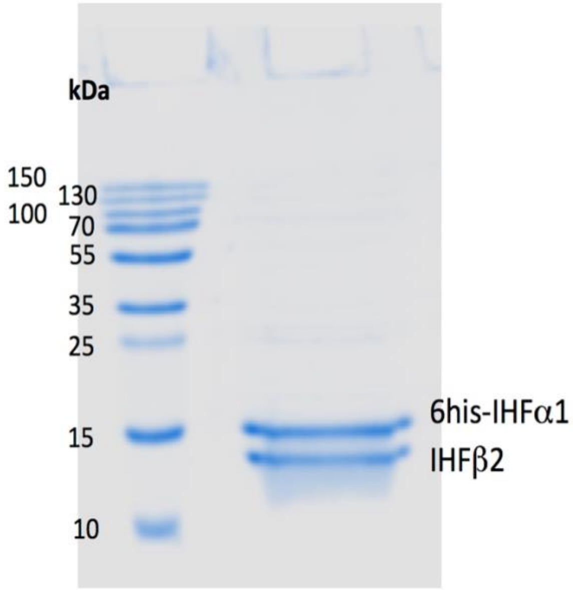

SUPPLEMENTARY FIGURE 5 IHF heterodimer purified with Ni-NTA agarose (Qiagen).

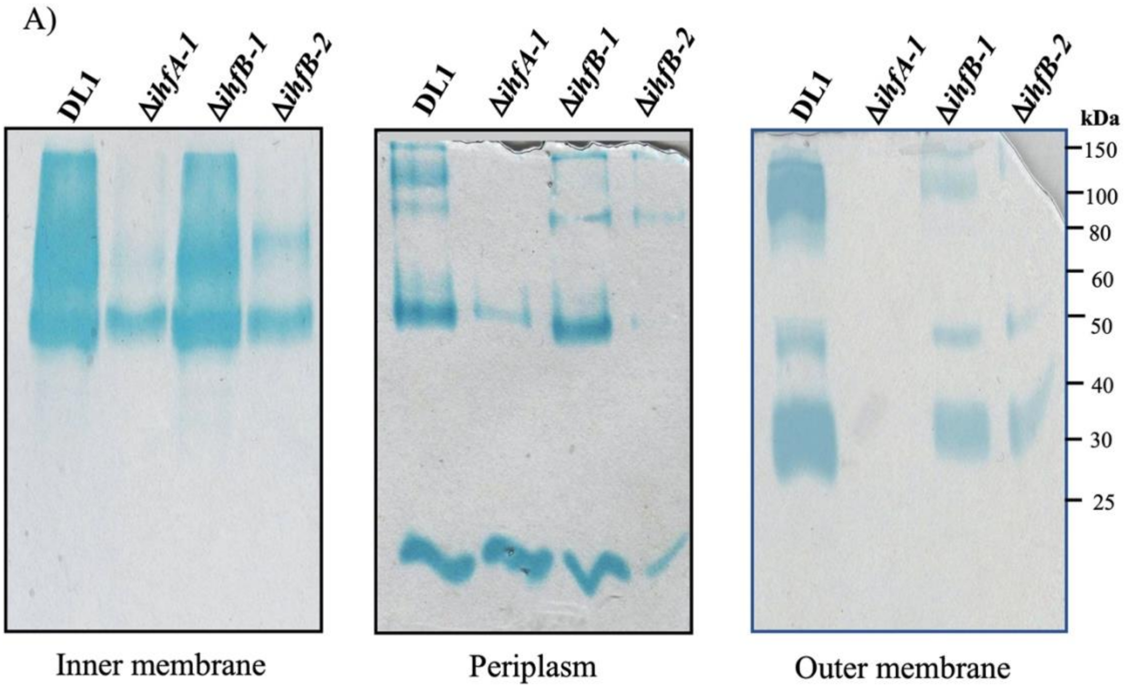

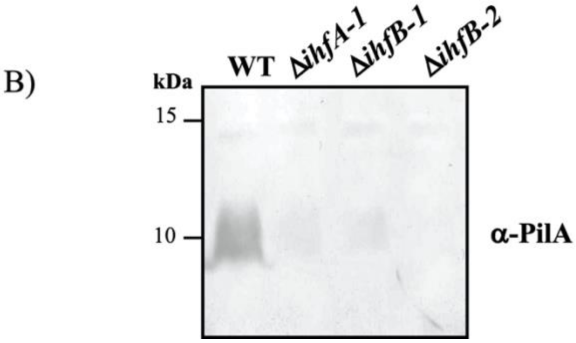

Full scan of the entire original gel shown in **FIGURE 5.**
